# A randomized controlled trial of a preventive intervention for the children of parents with depression: mid-term effects, mediators and moderators

**DOI:** 10.1186/s12888-023-04926-2

**Published:** 2023-06-21

**Authors:** Johanna Löchner, Belinda Platt, Kornelija Starman-Wöhrle, Keisuke Takano, Lina Engelmann, Alessandra Voggt, Fabian Loy, Mirjam Bley, Dana Winogradow, Stephanie Hämmerle, Esther Neumeier, Inga Wermuth, Katharina Schmitt, Frans Oort, Gerd Schulte-Körne

**Affiliations:** 1 Department of Child and Adolescent Psychiatry, Psychosomatics and Psychotherapy, University Hospital, LMU Munich, Munich, Germany; 2 Department of Child and Adolescent Psychiatry, University Hospital, Eberhard-Karls-University, Tübingen, Germany; 3 Human Informatics and Interaction Research Institute (HIIRI), National Institute of Advanced Industrial Science and Technology (AIST), Tsukuba, Japan; 4 Faculty of Social and Behavioral Sciences, Universiteit van Amsterdam, Amsterdam, Netherlands

**Keywords:** Prevention, Parental depression, Offspring, Family intervention, High-risk

## Abstract

**Background:**

In a parallel randomized controlled trial the effectiveness of the family- and group-based cognitive-behavioural “Gug-Auf” intervention in preventing depression in children of depressed parents was evaluated. We hypothesized that the intervention would be associated with reduced incidence of depression at 15 months as well as with reduced symptom severity at 6, 9, and 15 months. We also explored the role of a number of mediators and moderators.

**Methods:**

Families were included if a parent (n = 100, mean age = 46.06, 61% female) had experienced depression and children (n = 135, aged 8–17 years, 53% female) had no mental illness. Families (91.5% German) were randomly allocated (50:50 block-wise; stratified by child age and parental depression) to the 12-session “GuG-Auf” intervention or no intervention. Outcomes were assessed (on an intention-to-treat basis) at 0-(T1), 6-(T2), 9-(T3) and 15-months (T4) after baseline. Primary outcome (onset of depression; T4) was assessed with standardized (blinded) clinical interviews. Secondary (unblinded) outcome was risk of depression (at T2-T4) indicated by self- and parent-reported symptoms of internalizing, externalizing and depressive disorder. Potential mediators were emotion regulation, attributional style, knowledge of depression and parenting style. Potential moderators were parental depression severity and negative life events.

**Results:**

None of the children who received the intervention developed depression, whereas two of those in the control group did. The intervention significantly reduced depression risk (indicated by severity of self-reported internalizing symptoms) at T3 (*p* = .027, *d* = -0.45) and T4 (*p* = .035, *d* = -0.44). Both groups showed reduced depressive symptoms (*p* = .029, *d* = -0.44). Cognitive problem-solving and negative parenting emerged as mediators. There was no evidence that the intervention was associated with parent-reported internalizing symptoms or externalizing symptoms. No adverse events were observed.

**Conclusions:**

Children of parents with depression showed an increase in self-reported (but not parent-reported) internalizing symptoms over time. This increase was not present in children who received the GuG-Auf intervention. The intervention was not associated with changes in externalizing symptoms. Conclusions regarding prevention of the onset of depression were not possible. Despite some limitations in the generalizability, these findings contribute to reducing the burden of youth depression.

**Registration:**

The trial was registered on 16/04/2014 at ClinicalTrials.gov (NCT02115880) and study protocol published in BMC Psychiatry (https://bmcpsychiatry.biomedcentral.com/articles/10.1186/s12888-014-0263-2).

**Supplementary Information:**

The online version contains supplementary material available at 10.1186/s12888-023-04926-2.

## Background

### Parental depression as risk factor for depression

Having a parent who has experienced depression is one of the biggest risk factors for developing depression: children with a parent who has experienced depression have around a 40% likelihood of becoming depressed by the age of 20 [1] which is around three times the risk faced by children of parents without mental illness [2]. Children of parents with depression also experience more severe and chronic courses of disorder [3]. Integrative models [4, 5] propose that depression risk is conferred not only via genetic and neurobiological pathways [6] but also via increased exposure to adverse life events [7], family discord [8] and maladaptive parenting [9]. The combination of these effects is thought to hamper children’s cognitive and emotional processing [10] and ultimately their ability to cope with stress [11]. Interventions which target modifiable risk and protective factors are therefore likely to show promise in the prevention of depression.

### Preventive intervention for children of depressed parents

A number of psychological interventions have been developed to prevent depression in children of parents with depression. These preventive interventions target the aforementioned pathways to inter-familial depression: madadaptive parenting, increased stress exposure and cognitive and affective vulnerabilities to stress [4]. Some interventions focus on improving *stress resilience* using techniques from cognitive-behavioural therapy (e.g., the Coping With Depression intervention [12]). Other interventions address the *parenting difficulties* many parents with depression face [13]. Family-based interventions such as the Family Talk Intervention (FTI) have focussed on improving *family communication* via improved knowledge about the effects of parental depression [14]. One intervention which combines all of the aforementioned aspects is the Family Group Cognitive-Behavioural (FGCB) intervention [15]. The FGCB consists of eight weekly followed by four monthly sessions, each of which is run by two mental health professionals. Preliminary sessions address families’ knowledge about depression whereas subsequent sessions, conducted with parents and children separately, teach parenting strategies to parents and coping strategies for dealing with stress in children. Indeed, improvements in both parenting and children’s coping with stress have been shown to mediate the effects of the FGCB intervention on risk of depression [16]. As far as we are aware, this is the only trial to investigate the potential mediators of a preventive intervention for children of parents with depression. More frequently investigated are the factors which *moderate* the effects of preventive interventions for the offspring of parents with depression. For example, some [12, 17], but not all [18] trials have found more favourable effects of preventive interventions for families where the parent was remitted (rather than currently depressed) at baseline.

A meta-analysis of seven RCTs evaluating preventive interventions for children of parents with depression found small but significant effects on children’s self-reported internalizing symptoms immediately after the intervention (*g*′ = −0.20) and moderate effects the incidence of depression (k = 4, risk ratio = 0.56) [19]. However, the effectiveness of preventive interventions for children of depressed parents beyond 12 months has only been investigated in five trials [12, 14, 18, 20, 21]. Interestingly, in two trials the positive effects of the intervention only emerged 9- [12] and 12-months [18] after baseline. This contradicts the premise that intervention effects naturally may weaken over time and may reflect the fact that the benefit of acquired coping strategies only becomes apparent when stressful events are encounterered. An additional limitation of previous research is that just four trials have investigated the effectiveness of preventive interventions on the incidence of depression [12, 14, 18, 20], with the remainder using symptom severity as a proxy for depression risk.

The trial reported in this manuscript seeks to evaluate the effectiveness of the FGCB in Germany (see Study Protocol [22]). The FGCB [15] was adapted to German language and culture and abbreviated to GuG-Auf (“Gesund und glücklich aufwachsen”). Cultural adaptations included the formality with which parents were addressed (using the German “Sie” and their surnames rather than first names) and referring to locally appropriate leisure activities (e.g. football rather than baseball) and food (e.g. pretzels instead of crisps). A qualitative evaluation revealed generally high levels of acceptance of GuG-Auf [23]. GuG-Auf has also been shown to have positive effects on self-reported internalizing and externalizing symptoms directly after the intervention [24]. However, it remains to be tested whether the positive effects on symptoms are maintained in the long-term, which factors mediate and moderate intervention effects, and whether GuG-Auf has an effect on the incidence of depression.

### The current study

In this parallel randomized controlled trial 100 families were randomly allocated to either the experimental group (EG), who received GuG-Auf, or the control group (CG), who received no intervention. We conducted assessments at 6- (T2) 9- (T3) and 15- (T4) months after baseline (T1). A previous manuscript reported effects in the short-term (T2) using data from the oldest child from each family [24]. The current manuscript reports findings from all four time points (T1-T4), from *all* children within each family, and includes both changes in symptom severity and the *incidence of depression* (T4). Furthermore, path models directly test whether the effect of the intervention on secondary outcomes is mediated by children’s knowledge of depression, attributional style, coping strategies and parents’ parenting.

The analyses reported here are based on apriori hypotheses generated prior to commencing the trial and independent of the analyses of the T1-T2 data [24]. Since the original trial of FGCB found a significantly lower incidence of depression at 24 months in the EG (13%) versus the CG (26%) [18], our *first* and primary hypothesis was that at T4, more children in the CG would have a diagnosis of depression than in the EG. Since multi-finality approaches to developmental psychopathology suggest that individual interventions may have further-reaching effects on multiple outcomes [25] we also assessed whether any other mental illnesses were present since the beginning of the study.

Positive effects of the FGCB on self-reported symptoms of depression and externalizing symptoms (proxies for depression risk) only emerged 12-months after baseline [18], hence our *second* hypothesis was that at T3 and T4, children in the CG (versus EG) would show increases in our secondary outcomes of self-reported and parent-reported internalizing and externalizing symptoms as well as self-reported symptoms of depression. We expected an increase in symptoms over time in the CG and either no change or a reduction in the EG. Although we had originally intended to treat all secondary outcomes equally [22], prior to data analysis we created a hierarchy. In order of importance we assessed intervention effects on self-reported (i) internalizing, (ii) externalizing, and (iii) depressive symptoms and well as parent-reported (iv) internalizing and (v) externalizing symptoms at T1-T4. We prioritized child self-report measures over parent-reports due to poor correlations between parents with depression and their children [26] and because a previous study had found larger effects on self-reported versus parent-reported symptoms [15]. Parent-reports were nevertheless included in order to enhance the outcome validity of the findings [27]. We prioritized symptoms of internalizing disorder over symptoms of depression because the chosen measure of depressive symptoms (DIKJ) showed relatively poor reliability in our sample [24] and because the original FGCB trial found stronger effects across broad symptoms of psychopathology rather than on depressive symptoms specifically [15].

Our *third* hypothesis was that group differences in symptom severity (internalizing, externalizing and depressive symptoms) at T3 and T4 would be mediated by three key pillars of the FGCB intervention (measured at T2 and T3): improving knowledge of depression in families, enhancing adaptive parenting strategies (providing warmth and structure) and teaching children coping strategies for dealing with stress. Indeed, parenting style and children’s coping with stress had mediated the effects of FGCB previously [16].

Due to heterogenous previous findings [12, 17, 18], our *fourth* aim was to explore whether the effect of the intervention on symptoms (internalizing, externalizing and depressive symptoms) at T3 and T4 was moderated by parental depression at T1. Since the intervention is designed to improve children’s resilience to stress, we also explored whether the experience of stressful life events during the 15-month time period moderated the effect of the intervention on symptoms of psychopathology.

Finally, in line with previous studies [28], our *fifth* aim was to explore whether parents in the EG showed a greater reduction in symptoms of depression at T4 than parents in the CG.

## Methods

### Transparency and openness promotion (TOP)

The authors confirm that the manuscript meets the TOP, JARS and CONSORT guidelines. The study was pre-registered on 16/04/2014 with ClinicalTrials.gov (https://www.clinicaltrials.gov/ct2/show/NCT02115880) and the protocol published [22]. We report how we determined our sample size, all data exclusions, all manipulations, and all measures in the study. The data (excluding variables which may identify people) and analysis scripts are available on the Open Science Framework (https://osf.io/q7b6r/). The intervention materials will be made available to readers upon reasonable request.

### Study design

**Fig. 1:**
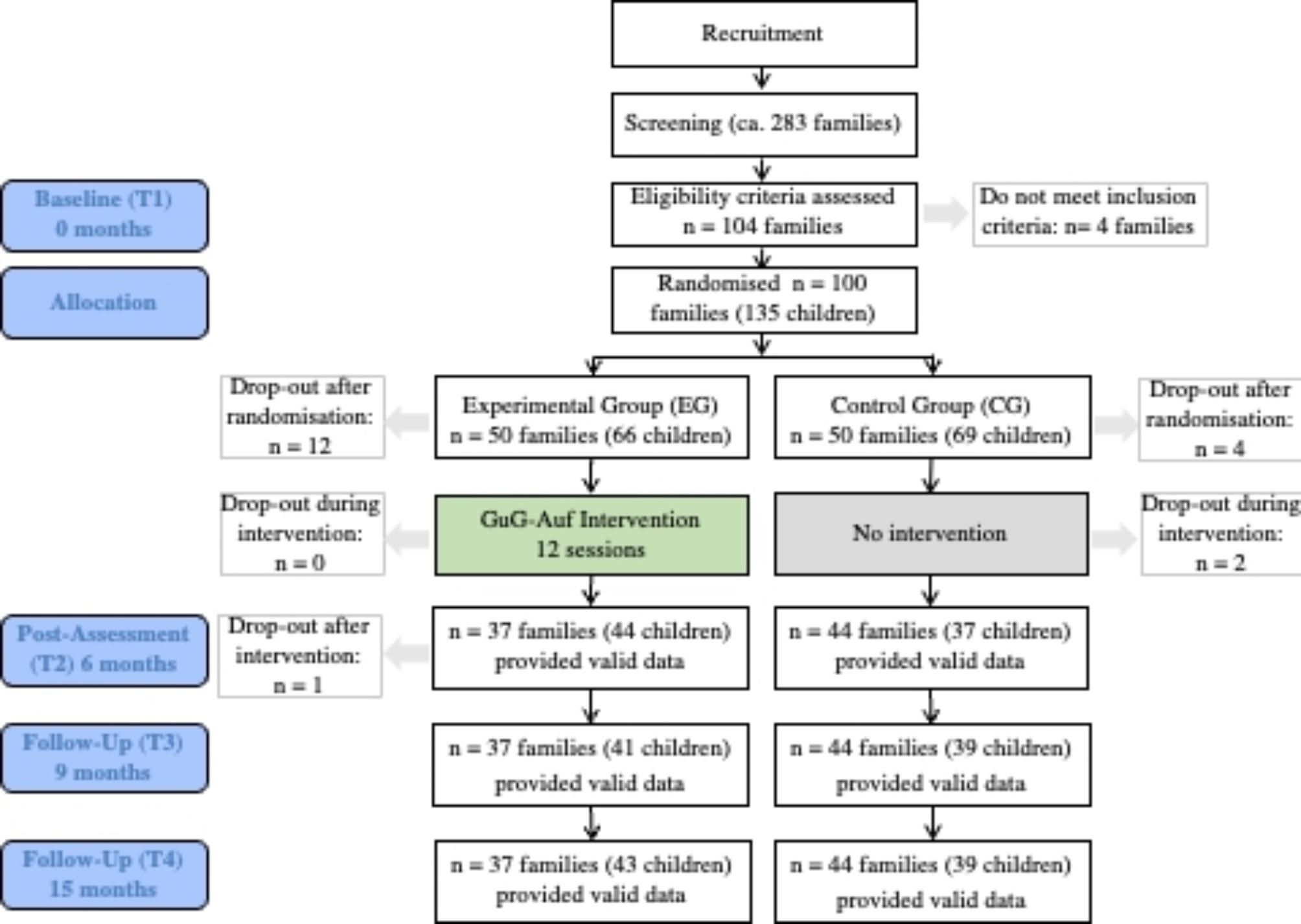
Study design and participant flow

This parallel randomized controlled trial (Fig. 1) evaluates a German adaptation (“Gesund und glücklich aufwachsen”; GuG-Auf) of the FGCB intervention [15]. JL and KW-S enrolled families and BP allocated them to either the experimental group (EG) or control group (CG). The randomization procedure was carried out by an independent researcher (FO) who was blinded by the identity of the families. The randomization sequence was computer-based and generated by an independent researcher (FO) who sent the coded randomization results via email to the study leader (BP). The randomization was conducted in blocks (per 10 families recruited) and was stratified according to whether the parents were currently depressed (as opposed to in remission) and the age of the children. All families were evaluated at baseline (T1), immediately after the intervention (T2; 6 months), and nine (T3) and fifteen months (T4) after the start of the study. In the single-blind study, participants knew about the assigned group, but the final outcome assessors (clinical interviews conducted at T4) did not know about the group assignment, and participants were strictly asked not to inform the final outcome assessor about their assignment. When calculating the necessary sample size we first examined previous studies which had also adopted an inactive control condition. These suggested that across the fifteen-month time period we could expect roughly 33% of the CG to encounter an episode of depression [29, 30]. We then examined the incidence of depression in the original trial of the FGCB, which suggested that we could expect roughly 10% of the EG to encounter an episode of depression across the same time period [14]. This difference equates to an Odds Ratio of 4.43. A sample size calculation for a one-sided Fisher’s exact test revealed a necessary sample size of 92, assuming a power of 80% and a 5% alpha level. The necessary sample size was exceeded (*n* = 100). Full details of the randomization procedure and sample size calculation are provided elsewhere [24].

### Participants and procedure

The study was conducted at the Department of Child and Adolescent Psychiatry, Psychosomatics and Psychotherapy at the LMU University Hospital (Germany). Families were recruited (largely through public advertisements) between July 2014 and October 2017. Recruitment of the sample is described in more detail elsewhere [24]. Follow-up data were collected between August 2015 and February 2019. One hundred families (EG = 50, CG = 50) with 135 children (EG = 66, CG = 69) were included in the study if one parent met the Diagnostic and Statistical Manual of Mental Disorders (DSM-IV) [31] criteria for a depressive disorder during the children’s lifetime and the child(ren) (8–17 years, IQ > 85) did not meet the diagnostic criteria for a psychiatric disorder in the present or past. Siblings who did not meet the study criteria (e.g. too young) were allowed to participate in the intervention as long as they had no acute mental health problems which would interfere with the intervention. The participants had to speak fluent German. Parents were excluded if the diagnostic interview revealed that they suffered from alcohol or drug abuse, a bipolar disorder, reported psychotic symptoms, had a personality disorder or were in a suicidal crisis. If both parents suffered from depression, both parents were entitled to the intervention. Families who participated in family-based therapy which could affect the effects of the intervention were excluded. In total four families were excluded following baseline assessment (three due to children fulfilling criteria for a DSM-IV disorder and one due to parent fulfilling exclusion criteria). Following a telephone screening, families attended the laboratory to provide informed consent and participate in clinical interviews. Questionnaires were handed out, which they could take home and return within a week. Once 10 families were recruited, a randomization process was carried out and families were informed which group they had been assigned to. Questionnaires at T2-T4 were delivered by post. At T4 (15 months) families were invited back to the laboratory for clinical interviews. Each family received €25 at the beginning and end of the study period as compensation for their time. All participants were informed about the course of the study and possible risks and gave their written consent to participate in the study. The study was approved by the Ethics Committee of the Medical Faculty at the University of the LMU Munich (Study ID: 3–14) and conducted in accordance with the Declaration of Helsinki.

**Table 1:** Demographic Characteristics of Children and Parents at Baseline

	EG	CG	Total
**Children**	***n*** = 66	***n*** = 69	N = 135
Age, mean (SD)	11.41 (2.70)	11.76 (2.95)	11.59 (2.83)
Gender (%) female	50.8	55.2	53.0
IQ, mean (SD)	105.40 (16.23)	106.46 (13.69)	105.96 (14.90)
Siblings (%)	77.8	72.7	75.3
School type (%)			
Primary school	37.5	35.2	36.4
Hauptschule	5.4	1.9	3.6
Realschule	12.5	11.1	11.8
Gymnasium	42.9	50.0	46.4
**Parents**	***n*** **= 50**	***n*** **= 50**	** N = 100**
Age, mean (SD)	45.15 (5.80)	47.10 (7.01)	46.06 (6.43)
Gender (%) female	60.0	62.7	61.4
Highest level of education (%)			
High school	14.0	18.2	15.8
A-levels	23.3	30.3	26.3
University	46.5	51.5	48.7
Doctoral degree	16.3	0	9.2
Family income (%)			
< €2000 /month	10.3	12.5	11.3
€2000 – €3000 /month	17.9	18.8	18.3
€3000 – €4000 /month	15.4	18.8	16.9
€4000 – €5000 /month	30.8	25.0	28.2
> €5000 /month	25.6	25.0	25.4
Depressive Symptoms (BDI-II)	16.7 (10.04)	17.7 (12.29)	17.20 (11.10)
Currently depressed (%)	58.0	56.9	57.4
Treatment experience			
Psychotherapy (%)	92.3	94.3	93.2
Psychopharmaceuticals (%)	82.1	69.7	76.4

*Note.* BDI-II = Beck’s Depression Inventory

### Sample description

Table 1 describes demographic characteristics of the children included in the study, the characteristics of parents are described elsewhere [24]. Most families lived in Munich and its suburbs and showed a comparatively high socio-economic background (49% of parents had a university degree and 54% of families earned over €4000 per month). Most parents were diagnosed with recurrent depressive disorder of mild (64.5%) or moderate (12.5%) severity, 23% were in remission. 10% fulfilled the criteria for a double depression (experiencing episodes of major depression in addition to dysthymia). Only 14.8% had experienced a single depressive episode in their lifetime. 38% had comorbid diagnosis (mostly anxiety or eating disorders), 15% had slightly increased values on the personality disorder screening questionnaire (SKID II), but none showed clinically significant symptoms^1^. 11.5% of the families consisted of two parents suffering from depression. The partner of parents with depression, who reported not to be affected by a mental illness, was also screened for psychopathological impairment using the SCL-90-R indicating 11% with current symptoms of a psychiatric disorder.

### Measures

A more detailed description of the measurement instruments (including their psychometric properties in the current sample) is provided elsewhere [24].

#### Eligibility criteria

The semi-structured Diagnostic Interview for Psychiatric Disorders (DIPS) [32] was conducted with parents to assess whether they met study inclusion criteria regarding the presence of mental illness. The child version (K-DIPS) [33], which includes separate interviews with the child and parent, was used to ensure that children had no current or past mental illness. Where there was disagreement between parents and children, more weight was given to the child report. The short version (56 items) of the Culture Fair Test 20-R [34] was used to screen out children who had IQ < 85.

#### Primary outcome measure

The primary outcome of the study (Hypothesis 1) was the onset of depression since study begin which was measured at T4 using to the K-DIPS. We also coded whether any other mental illnesses were present since the beginning of the study.

#### Secondary outcome measures

Secondary outcomes measured the effect of the intervention on children’s *risk* of depression (Hypothesis 2). Risk of depression was measured, in order of importance, by self-reported (i) internalizing, (ii) externalizing, and (iii) depressive symptoms and well as parent-reported (iv) internalizing and (v) externalizing symptoms at T1-T4. Symptoms of depression were measured using the German version of the Children’s Depression Inventory (DIKJ) [35]. Children’s internalizing and externalizing symptoms were assessed using the German version of the Child Behavior Checklist (CBCL) and the equivalent Youth Self-Report (YSR) [36, 37]. As previously mentioned, we intended to treat all outcomes equally. To aid interpretation of the findings, prior to data analysis we assigned the aforementioned hierarchy to these secondary outcomes. We prioritized child self-report measures over parent-reports due to poor correlations between parents with depression and their children [26] and because a previous study had found larger effects on self-reported versus parent-reported symptoms [15]. Parent-reports were nevertheless included in order to enhance the outcome validity of the findings [27]. We prioritized internalizing symptoms over symptoms of depression specifically, because the chosen measure of depressive symptoms (DIKJ) showed relatively poor reliability in our sample [24] and because the original FGCB trial found stronger effects across broad symptoms of psychopathology rather than on depressive symptoms specifically [15]. This is in line with multi-finality approaches to developmental psychopathology [25].

#### Mediators

To test which factors mediated the effects of the intervention on symptoms of psychopathology (Hypothesis 3), four measures were used. These measures were selected based on their psychometric properties and availability in German language and were broadly designed to match the key pillars of the intervention: improving knowledge about depression, teaching positive parenting skills, teaching coping (emotion regulation) strategies to children. Children’s *knowledge of depression* was assessed using the German “Depression Knowledge Questionnaire” [38]. Children’s *emotion regulation* (ER) was measured using the Emotion Regulation Questionnaire for Children and Adolescents (FEEL-KJ) [39] which evaluates how children deal with the emotions fear, sadness and anger. It assesses the use of seven adaptive (problem-solving, distraction, positive thinking, acceptance, forgetting, reappraisal, cognitive problem-solving) and five maladaptive (giving up, aggressive actions, withdrawal, self-devaluation, rumination) ER strategies. Full descriptions of the strategies including the items used to measure them and how they map onto the so-called A-APP coping strategies taught in the GuG-Auf intervention are provided in the Supplementary Table S1. The sum scores for adaptive and maladaptive strategies were calculated for each emotion (anger, fear and sadness). Because a primary strategy taught to children was cognitive reappraisal, we included an measure of *attribution style* specifically: the Attribution Style Questionnaire (ASF) for Children and Adolescents [40]. We used sum scores of the three dimensions of the positive and negative attribution scales. *Parenting style* was measured using the Inventory of Parenting Styles (ESI) [41] which children completed about the parenting style of their parents. Sum scores were calculated for positive and negative parenting styles.

#### Moderators

To explore whether parental depression moderated the effect of the intervention on children’s symptoms of psychopathology we used the German version of the 21-item Beck’s Depression Inventory (BDI-II) [42] at T1. To explore the moderating role of children’s life events we used the *Child and Adolescent Survey of Experiences Parent and Child version* (CASE) [43] at T4. Scores for the number and the impact of positive as well as negative life events were calculated for self- and parent-report.

#### Parent mental health

To measure the effect of the intervention on parent mental (Aim 5) we re-administered the BDI-II [42] at T2-T4.

### GuG-Auf intervention

Participants in the EG received the group-based GuG-Auf intervention (manual available upon request). Eleven groups with 3–5 families were conducted between January 2015 and June 2018. The sessions took place in the Department of Child and Adolescent Psychiatry, Psychosomatics and Psychotherapy. The contents of the manual are described in more detail elsewhere [24]. The intervention takes place over six months and contains 12 sessions (eight weekly followed by four monthly) each two hours long. Of the families who attended at least one session, the average number of sessions attended was nine. There are sessions for the whole family as well as separate sessions for parents or children only. The intervention is based on three components: Psycho-education about depression (parents and children), coping strategies for dealing with stress for children (A-APP strategies: acceptance, distraction, positive thinking and positive activities) and parenting training for parents (parenthood and depression, showing warmth and structure). Children and parents have homework to do between sessions, and parents are encouraged to spend at least 15 min per week of quality time with their children. 69.8% of children and 60.6% of parents completed the homework assigned to them. Each session is led by two group leaders who are either psychology graduates or child and adolescent psychiatrists in training. All group leaders were trained in using the manual and supervised on a regular basis by the principal investigators. To assess fidelity with the manual, video recordings of 25% of the sessions were compared with an intervention checklist. 98% of the sessions were fully completed (range 87–100%). The fidelity of the intervention is described in more detail elsewhere [24].

### Control condition

Participants in the CG did not receive any intervention but, like those in the EG, were entitled to receive support from the usual health-care system (e.g. parent counselling centres, family doctor). Participants of the CG were offered the intervention as a written document after completing T4 (15 months from baseline). Anecdotally, families in both groups reported that they received support from their family doctor or counselling centres, although this was not systematically evaluated.

### Strategy of the analysis

The data was analysed using SPSS version 19 (SPSS Inc., 1989–2006) and R [44] for Windows. To assess the extent to which outcomes measures correlated between children and parents belonging to the same family (i.e., assumption of independence) intra-class correlations were calculated. Since these values were low (variance explained < 0.35 for all outcome measures), we did not include family membership in the statistical models. We modelled family-specific variance in the moderator analyses because parental depression relates to every family but not to every child.

#### Hypothesis 1: effects of the intervention on the onset of depression at T4

Our intention was to use logistic regression models to calculate Odds Ratios for depression diagnosis (our primary outcome). However, data were only available for 31 children (44.93%) in the CG and 32 (48.48%) in the EG. The frequencies of any psychiatric disorder were so low in both the EG (*n* = 2; 6% of those who provided data) and CG (*n* = 5; 16% of those who provided data), that a statistical analysis of these data was not possible.

#### Hypothesis 2: effects of the intervention on symptom severity at T2, T3 and T4

We used multilevel modelling (MLM) to test the effect of the intervention on the five measures of symptom severity (see “Secondary Outcome Measures”). Each of the outcome measures was predicted by the treatment group (dummy-coded with CG as 0 and with EG as 1), time variables (i.e., DT1, DT2, and DT3 coded as 1 for T2, T3, and T4, respectively), and the group-time interactions. Due to most variables being skewed (see Supplemental Table S3), the outcome measures were log-transformed prior to the model estimation. We assumed random effects for the intercept and time dummies to allow the parameters vary across individuals unless there were any convergence problems. To calculate effect sizes (Cohen’s *d*) the interaction effects of group x time were divided by the pooled SD from the two groups at T1 [45–47]. A within-group change was defined as a simple slope of a time variable for each group – equivalent to a paired t-test [48, 49]; the effect size *d* was then defined as the within-group change, divided by the SD from the respective group at T1 [46].

**Marginal means and SEs.** Missing values were handled by the ML estimation [50] assuming data were missing completely at random (MCAR) [51]. To inspect the pattern of missingness, we tested the correlations between the T1 scores for each variable and the number of assessments that each child completed. There were no significant correlations with any of the symptom measures (|rs| < 0.11, *p*s > 0.28).

#### Hypothesis 3: potential mediators of the intervention effects at T2, T3 and T4

The same MLM approach reported above was used to estimate which of the four potential mediators (see “Mediators”) showed evidence of group-dependent change over the four time periods. Four outcomes were modelled: (i) coping with stress (FEEL-KJ; 12 individual strategies and the two sub-scales adaptive and maladaptive ER), (ii) attributional style (ASF; 6 sub-scales), (iii) knowledge of depression (Knowledge of Depression questionnaire) and (iv) parenting style (ESI; positive and negative styles). We tested the correlations between the T1 scores for each variable and the number of assessments that each child completed. Only two of the potential mediator outcome variables showed a correlation with the amount of missing values at T1: the FEEL-KJ subscales acceptance (*r* = .27; p = .01) and reappraisal (*r* = .24, p = .01). Since the outcome of MLMs for these variables was unchanged whether or not the number of completed assessments was controlled for we report estimates without the control.

Outcomes which showed group-dependent change over time in the MLM (the “a path” in Baron-Kenny formulation [52]) were included in structural equation modelling (SEM), assuming a lagged effect of a mediator on an outcome variable: i.e., the dual simplex model [53]^2^. Specifically, (a) the mediator score at the current time point, t, was predicted by the treatment group after controlling for the score at the previous time point, t-1; and (b) the outcome at time t was modelled by the treatment group and the mediator at t-1 after controlling for the outcome score at t-1. We did not assume treatment effects on the mediator or outcome at T4 because of the large temporal distance. This model allowed for testing indirect effects via different pathways leading to the outcome at T4. Model fit was assessed using the Comparative Fit Index (CFI) and Root Mean Square of Approximation (RMSEA), indicating good fit with CFI > 0.90 and RMSEA < 0.05. The mediation models were estimated using the R package, lavaan [54] with the full-information maximum likelihood estimator to deal with missing values. All variables were standardized in order to adjust the differences in the variance across variables.

#### Fourth aim: exploration of parental depression and stressful life events as moderators of the intervention effects

The MLM models reported in Hypothesis 2 testing interactions between group (EG, CG) and time (T1-T4) were re-run to include the potential moderators (i) parental BDI-score at T1 and (ii) parental diagnostic status (currently depressed; yes/no) at T1. Similarly, moderating effects of negative life events (CASE) were operationalized by (i) the *number* child-reported negative life events, and (ii) the *impact* of negative life events at T4. Participants who had missing values for the moderators were excluded in the models.

#### Fifth aim: effect of the intervention on parental symptoms of depression

The same MLM models described in Hypothesis 2 were used to investigate the potentially beneficial effects of the intervention over time (T1-T4) on parental depression (BDI-II).

## Results

Figure 1 provides an overview of the participant flow within the trial. In total, 38 out of 50 (76%) randomized families completed the intervention. Twelve EG families withdrew after randomization, mostly for reasons of time (8) or because the child was affected by a psychiatric disorder (1). Some did not give reasons (2) or could not be contacted (1). One family (EG) discontinued the study after the intervention was completed because they had moved. Four families in the CG discontinued the study after randomization for unknown reasons (3), or because of marital disagreement about the risks of including their children in the study (1). Two families (CG) dropped out during the intervention period because the child no longer wanted to complete the questionnaires (1) or for unknown reasons (1). To our knowledge no severe adverse events occurred^3^.

### Missing data

Not all children completed all measures at all time points (see Fig. 1). High levels of missing data for the primary outcome (37%) meant that statistical analysis of this data was not possible (see Hypothesis 1: Treatment effects on depression onset). Supplemental Table S4 provides the frequency of missing values for the remaining outcome measures. 118/135 (87.41%) provided data on at least one of the secondary outcome (symptom measures) at least once across the four time points. 66/135 (48.89%), 30 EG, provided data at all four time points. 16/135 (11.85%), 9 EG, provided data at three time points. 8/135 (5.93%), 4 EG, provided data at two time points. 27/135 (20.0%), 15 EG, provided data at one time point. The amount of missing data did not vary significantly between groups at any of the time points^4^. We also tested the correlations between the T1 scores for each variable and the number of assessments that each child completed. As mentioned previously, missing values for the MLM analysis of secondary outcome measures and mediators were handled by the ML estimation [50] assuming data were missing completely at random (MCAR) [51]. Only two of the potential mediator outcome variables showed a correlation with the amount of missing values at T1: the FEEL-KJ subscales acceptance (*r* = .27) and reappraisal (*r* = .24). Since the outcome of MLMs for these variables was unchanged whether or not the number of completed assessments was controlled for we report estimates without the control.

### Hypothesis 1: treatment effects on depression onset (primary outcome)

Data for our primary outcome were available for 31 children (44.93%) in the CG and 32 (48.48%) in the EG. The frequencies of any psychiatric disorder were so low in both the EG (*n* = 2; 6% of those who provided data) and CG (*n* = 5; 16% of those who provided data) that the planned statistical analyses of these data were not possible^5^. Two children in the CG, versus no children in the EG, met criteria for depression at T4.

### Hypothesis 2: intervention effects on symptom severity (secondary outcomes)

Table 2 reports descriptive statistics for the secondary outcome variables internalizing and externalizing symptoms (self- and parent-report) and symptoms of depression (self-report) across the two groups and all four time points.

**Table 2:** Descriptive Statistics of Internalising, Externalising and Depressive Symptoms for Measurement Points T1, T2, T3 and T4

Variables	Experimental Group (*n*, *M, SD)*								Control Group (*n*, *M, SD)*											
	T1		T2		T3		T4		T1			T2			T3			T4		
Self-report symptoms																				
Internalising (YSR)	*54*	9.00 (7.69)	*42*	7.38 (7.82)	*40*	7.95 (8.03)	*40*	5.95 (5.97)		*40*	6.75 (6.20)	*31*	8.29 (7.4)	*35*		10.38 (9.17)	*32*		8.82 (9.12)	
Externalising (YSR)	*54*	8.85 (5.51)	*42*	8.52 (6.25)	*40*	7.63 (5.57)	*40*	7.65 (5.97)		*40*	8.82 (6.86)	*31*	9.84 (6.36)	*35*		8.91 (6.91)	*32*		8.97 (6.86)	
Depression (DIKJ)	52	7.83 (5.85)	*34*	8.59 (8.19)	*35*	6.86 (7.67)	*39*	5.64 (5.27)		*41*	7.56 (4.91)	*28*	6.46 (4.88)	*29*		5.38 (4.19)	*35*		5.60 (5.98)	
Parent-report symptoms																				
Internalising (YSR)	56	7.96 (6.46)	*38*	7.34 (8.35)	*41*	6.00 (6.48)	*39*	5.05 (5.63)		*43*	7.05 (6.09)	*33*	6.55 (7.06)	*32*		6.03 (4.80)	*37*		6.30 (5.52)	
Externalising (CBCL)	56	6.93 (6.47)	*38*	6.11 (5.94)	*41*	5.15 (4.98)	*39*	4.69 (5.42)		*43*	4.60 (5.70)	*33*	3.52 (3.86)	*32*		4.00 (4.12)	*37*		3.30 (3.19)	

*Note* CBCL = Child Behavior Checklist; DIKJ = Depressions-Inventar für Kinder und Jugendliche; YSR = Youth Self-Report

#### Child-reported internalizing and externalizing symptoms (YSR)

**Fig. 2:**
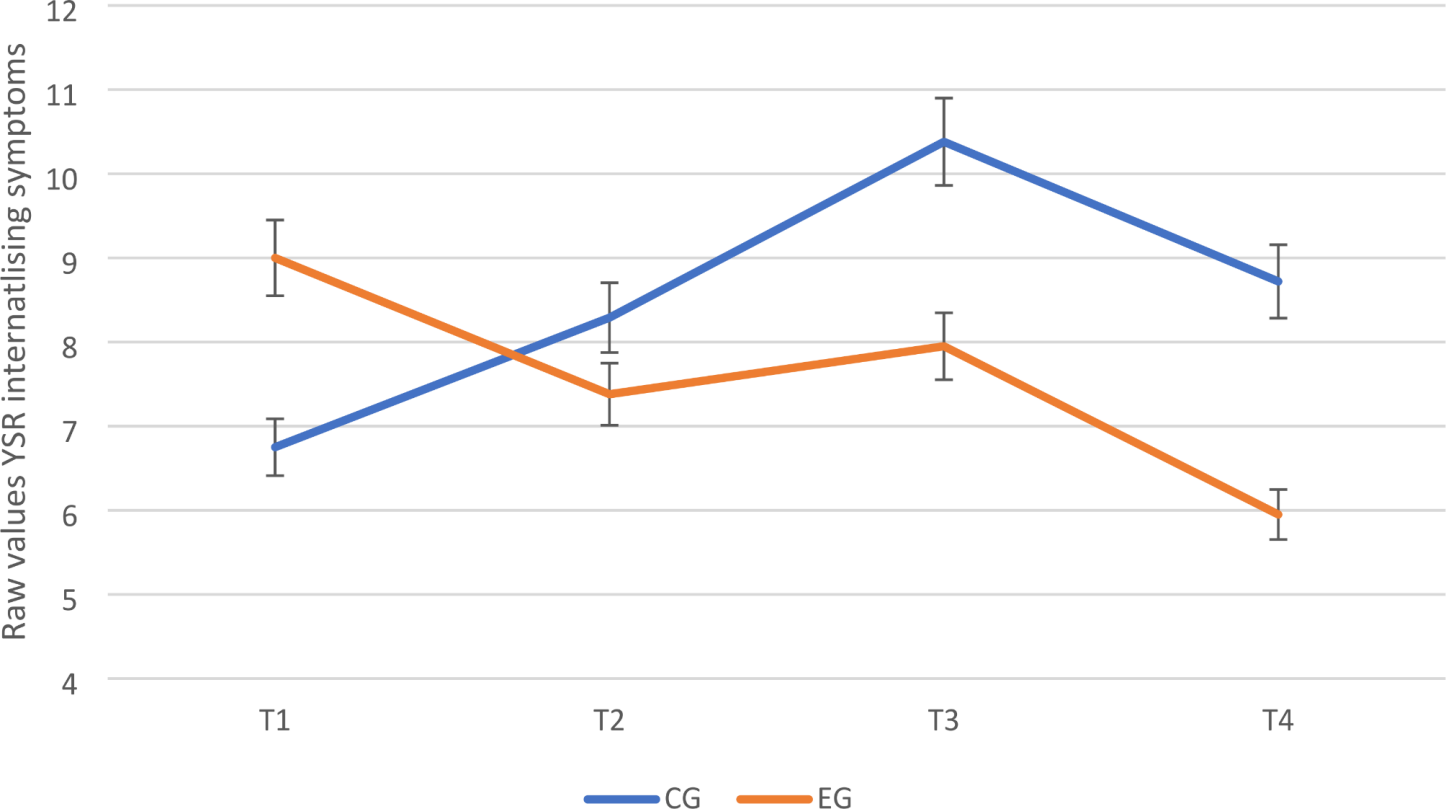
Changes in Self-Reported Internalising Symptoms Across Time of Children in the EG versus CG

As depicted in Fig. 2 there were significant interactions between group and time for self-reported internalizing symptoms from T1 to T3 (estimate = -0.42, *SE* = 0.19, *t* = -2.22, *p* = .027; *d* = -0.45, 95% *CI* [-0.84, -0.06]) as well as from T1 to T4 (estimate = -0.41, *SE* = 0.19, *t* = -2.12, *p* = .035; *d* = -0.44, 95% *CI* [-0.84, -0.05]) but not from T1 to T2 (*p* > .05). Simple slope (post-hoc) analyses revealed a significant increase from T1 to T3 in the CG (estimate = 0.36, *SE* = 0.14, *t* = 2.55, *p* = .012; *d* = 0.38, 95% *CI* [0.09, 0.67]) but no change in the EG (estimate = -0.06, *SE* = 0.13, *t* = -0.46, *p* = .647; *d* = -0.06, 95% *CI* [-0.32, 0.20]). From T1 to T4 the CG showed a non-significant increase (estimate = 0.18, *SE* = 0.14, *t* = 1.27, *p* = .208; *d* = 0.19, 95% *CI* [-0.09, 0.48]) whereas the EG showed a non-significant decrease over time (estimate = -0.22, *SE* = 0.13, *t* = -1.77, *p* = .080; *d* = -0.25, 95% *CI* [-0.51, 0.01]). No significant effects were found for self-reported externalizing symptoms (all *p*s > 0.05).

#### Self-reported depressive symptoms (DIKJ)

Both groups showed significant reductions from T1 to T3 (estimate = -0.30, *SE* = 0.14, *t* = -2.19, *p* = .029; *d* = -0.44, 95% *CI* [-0.64, -0.24]) and from T1 to T4 (estimate = -0.39, *SE* = 0.14, *t* = -2.75, *p* = .006, *d* = -0.57, 95% *CI* [-0.77, -0.37]), but not from T1-T2. The groups did not differ between each other in changes across time (*p* > .05).

#### Parent-reported internalizing and externalizing symptoms (CBCL)

There was no evidence of group differences in changes in internalizing or externalizing symptoms across time (all *ps* > 0.05).

### Hypothesis 3: treatment effects on the potential mediators

#### 3a. Group-dependent change in potential mediators

Descriptive data for the variables showing group-dependent change (Parenting Style and ER) are shown in Table 3. Descriptive data for the other variables (attributional style, knowledge of depression) are reported in Supplementary Table S2.

MLM revealed a significant group by time interaction for negative (but not positive) parenting style (ESI) between T1 and T3 (estimate: -6.36, *SE* = 2.15, *t* = -2.96, *p* = .004; *d* = -0.44, 95% *CI* [-0.82, -0.05]). This reflected a decrease in negative parenting in the EG (estimate = -4.43, *SE* = 1.48, *t* = -3.00, *p* = .003; *d* = -0.28, 95% *CI* [-0.55, -0.02]) and no change in the CG (estimate = 1.93, *SE* = 1.57, *t =* 1.23, *p* = .22; *d* = 0.15, 95% *CI*[-0.13, 0.42]). There were no group differences at any other time points for negative or positive parenting style.

Significant group by time interactions were found for one adaptive ER strategy (cognitive problem solving: Supplemental Figure S1)^6^ and two maladaptive strategies (self-devaluation: Supplemental Figure S2 and aggressive actions: Supplemental Figure S3).

The group by time interaction for cognitive problem-solving occurred from T1 to T2 (estimate = -2.63, *SE* = 0.99, *t* = -2.66, *p* = .010; *d* = -0.46, 95% *CI* [-0.84, 0.08]) and reflected *no change* in the EG (estimate = -0.88, *SE* = 0.66, *t* = -1.33, *p* = .187; *d* = -0.16; 95% *CI* [-0.42, 0.09]) and a significant *increase* in the CG (estimate = 1.75, *SE* = 0.74, *t* = 2.38, *p* = .019; *d* = 0.29; 95% *CI* [0.01, 0.56]). The group by time interaction in self-devaluation occurred from T1-T3 (estimate = -2.71, *SE* = 1.05, *t* = -2.58, *p* = .010; *d* = -0.53, 95% *CI* [-0.91, -0.15]) and reflected an increase in the CG (T1 to T3; estimate = 2.89, *SE* = 0.77, *t* = 3.75, *p <* .001; *d* = -0.61, 95% *CI* [0.31, 0.91]) and no change in the EG (*p* > .05). The significant interaction in aggressive behaviour occurred from T1 to T2 (estimate = 2.59, *SE* = 0.88, *t* = 2.93, *p <* .001; *d* = 0.66, 95% *CI* [0.27, 1.04]) and reflected an increase in the EG (estimate = 1.81, *SE* = 0.59, *t* = 3.07, *p <* .001; *d* = 0.50, 95% *CI* [0.23, 0.77]) and no change in the CG (*p* > .05).

**Table 3:** Descriptive Data on Changes in Emotion Regulation and Parenting Style in Both Groups Across all Four Time Points

	Experimental Group (*n*, *M, SD*)													Control Group (*n*, M, SD)															
	T1			T2		T3				T4				T1				T2				T3				T4			
Adaptive strategies (FEEL-KJ)	*57*	129.33 (31.19)		*43*	134.37 (33.78)	*40*		141.65 (34.67)		*40*		138.40 (35.04)		*40*		132.96 (28.48)		*35*		142.63 (27.31)		*38*		139.27 (24.39)		*38*		141.39 (30.94)	
Problem-solving	*57*	19.58 (5.00)		*43*	20.23 (5.24)	*40*		21.53 (5.26)		*40*		21.13 (5.71)		*48*		19.83 (5.35)		*35*		22.26 (5.41)		*37*		20.86 (5.30)		*38*		21.37 (5.6)	
Distraction	*57*	19.35 (5.99)		*43*	20.56 (6.22)	*40*		21.00 (6.07)		*40*		20.88 (5.74)		*48*		19.43 (5.36)		*35*		20.86 (4.91)		*37*		19.16 (5.97)		*38*		20.05 (5.20)	
Positive thinking	*57*	18.77 (5.95)		*43*	20.09 (5.69)	*40*		21.35 (5.82)		*40*		20.15 (6.17)		*48*		19.20 (5.76)		*35*		19.91 (5.83)		*37*		18.38 (6.12)		*38*		20.34 (5.21)	
Acceptance	*57*	17.77 (6.18)		*43*	18.81 (6.34)	*40*		20.60 (5.94)		*40*		20.35 (6.15)		*48*		19.60 (5.57)		*35*		20.00 (4.58)		*37*		21.29 (3.76)		*38*		20.68 (5.56)	
Forgetting	*57*	19.42 (4.98)		*43*	19.07 (5.09)	*40*		19.80 (5.1)		*40*		19.05 (5.32)		*48*		19.16 (4.66)		*35*		20.17 (4.46)		*37*		19.91 (3.90)		*38*		19.47 (4.86)	
Reappraisal	*57*	20.07 (5.31)		*43*	19.53 (5.28)	*40*		20.45 (6.1)		*40*		20.10 (6.11)		*48*		19.75 (6.11)		*35*		22.00 (5.19)		*37*		121.18 (5.34)		*38*		20.76 (5.75)	
Cognitive problem-solving	*57*	14.37 (5.33)		*43*	16.00 (5.31)	*40*		17.05 (5.86)		*40*		16.78 (5.84)		*48*		15.95 (4.67)		*35*		17.43 (5.20)		*38*		18.00 (3.89)		*38*		17.39 (5.04)	
Maladaptive strategies (FEEL-KJ)	*57*	69.35 (13.65)		*43*	71.63 (17.15)	*40*		71.43 (17.85)		*40*		67.58 (15.41)		*48*		69.68 (15.78)		*35*		72.03 (15.04)		*37*		76.13 (17.97)		*38*		70.45 (17.14)	
Giving up	*57*	13.89 (3.99)		*43*	13.93 (5.44)	*40*		14.53 (5.44)		*40*		12.85 (4.63)		*48*		13.06 (4.73)		*35*		13.11 (4.44)		*37*		14.45 (5.4)		*38*		13.61 (4.87)	
Aggressive actions	*57*	10.02 (3.62)		*43*	11.93 (4.04)	*40*		10.80 (3.50)		*40*		10.33 (3.56)		*48*		12.89 (4.63)		*35*		10.77 (4.06)		*37*		14.48 (5.47)		*38*		10.55 (3.64)	
Withdrawal	*57*	14.58 (4.39)		*43*	14.88 (5.36)	*40*		14.85 (5.81)		*40*		13.55 (4.67)		*48*		14.87 (4.80)		*35*		15.31 (4.8)		*37*		16.51 (4.63)		*38*		15.26 (4.64)	
Self-devaluation	*57*	13.68 (5.39)		*43*	13.67 (4.89)	*40*		13.68 (5.01)		*40*		13.58 (5.49)		*48*		13.68 (4.71)		*35*		15.09 (5.2)		*37*		16.94 (6.07)		*38*		14.95 (5.65)	
Rumination	*57*	17.18 (5.56)		*43*	17.21 (4.42)	*40*		17.53 (5.01)		*40*		17.28 (4.86)		*48*		16.95 (4.40)		*35*		17.74 (5.13)		*37*		16.81 (5.72)		*38*		16.08 (5.43)	
Positive Parenting (ESI)	*55*	74.71 (11.69)		*41*	74.51 (14.70)	*41*		73.21 (17.40)		*40*		69.65 (19.30)		*45*		70.51 (12.51)		*34*		72.00 (16.66)		*37*		65.27 (16.70)		*39*		67.95 (17.17)	
Negative Parenting (ESI)	*54*	67.50 (15.56)		*42*	64.62 (14.25)	*39*		60.82 (11.62)		*40*		65.35 (15.91)		*45*		67.11 (13.30)		*33*		67.70 (12.33)		*37*		69.35 (14.52)		*39*		69.85 (17.98)	

Children in both groups showed increases in their knowledge of depression over time (T1 to T2: estimate = 1.51, *SE* = 0.62, *t* = 2.41, *p =* .002; *d* = 0.38, 95% *CI* [0.18, 0.58]; T1 to T3: estimate = 1.60, *SE* = 0.82, *t* = 1.95, *p =* .005; *d* = 0.40, 95% *CI* [0.20, 0.60]; T1 to T4: estimate = 1.96, *SE* = 0.62, *t* = 3.17, *p <* .001; *d* = 0.49, 95% *CI* [0.29, 0.70]), but groups did not differ from each other. There was no evidence of group by time interactions in any of the attributional style (ASF) subscales (all *p* > .05).

#### 3b. Mediation models

**Fig. 3:**
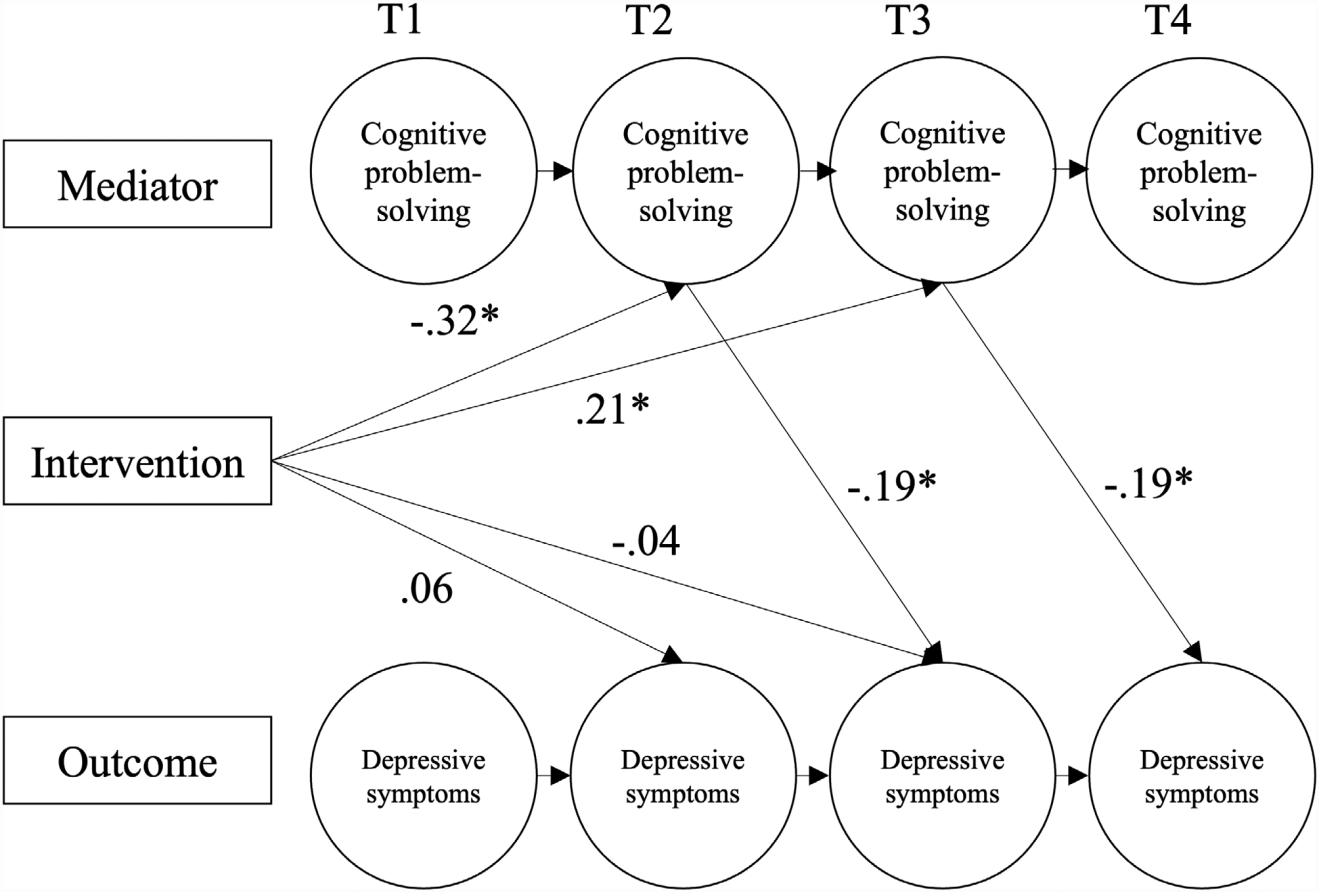
Path Diagram of the Dual Simplex Model for the Mediation Analysis with Cognitive Problem-Solving (FEEL-KJ) as Mediator on Depressive Symptoms (DIKJ). *Note*: Model fit indices: χ2(20) = 19.4, *p* = .50, CFI = 1.00, RMSEA < 0.001.

**Fig. 4:**
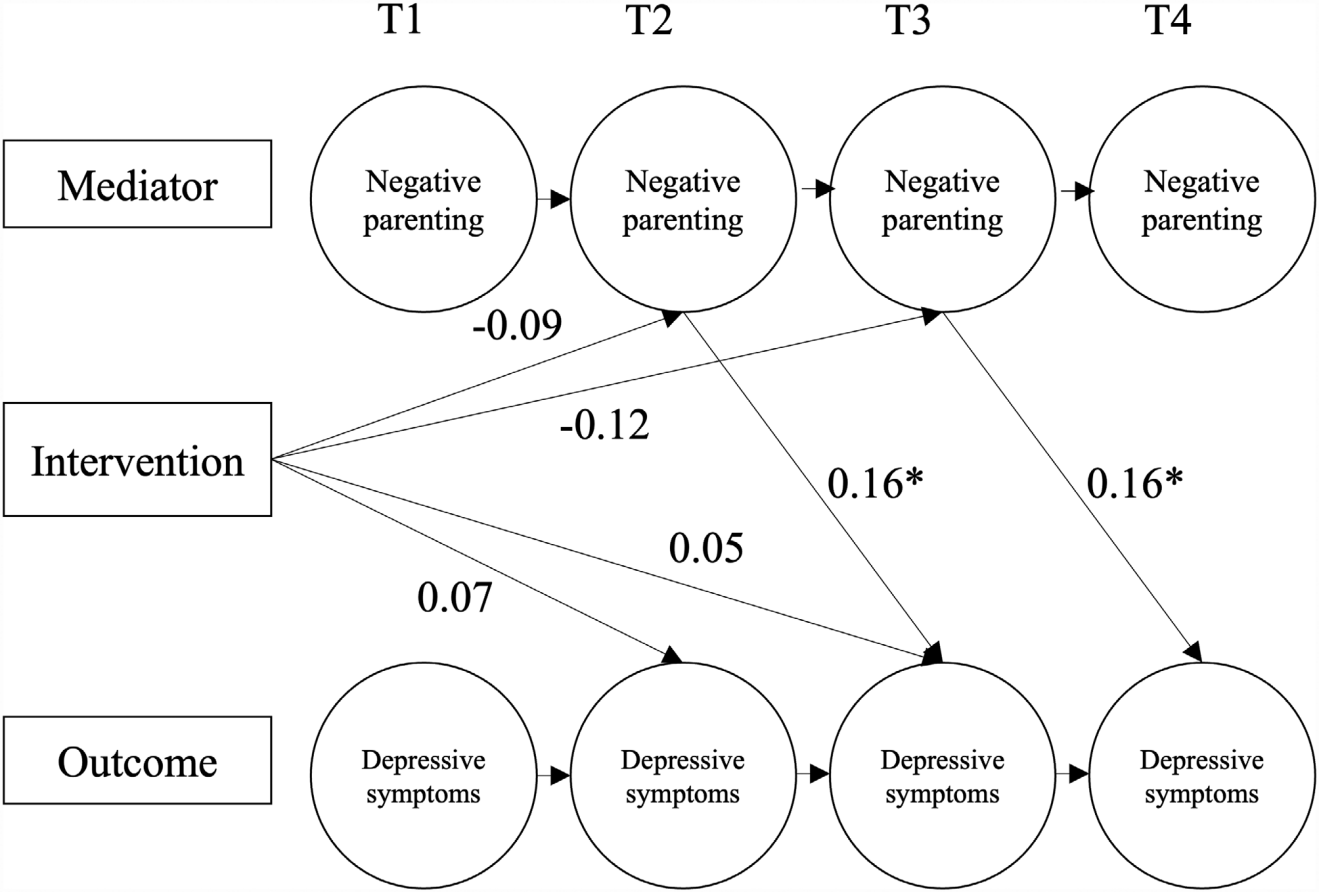
Path Diagram of the Dual Simplex Model for the Mediation Analysis with Negative Parenting (ESI) as Mediator on Depressive Symptoms (DIKJ). *Note*: Model fit indices: χ2(20) = 18.85, *p* = .53, CFI = 1.00, RMSEA < 0.001.

We estimated the dual simplex models with the candidate variables from phase *3a* ER (cognitive problem solving, self-devaluation, aggressive action) and negative parenting (ESI) as mediators and the five symptom measures as outcomes^7^. There were no significant paths found for child- (YSR) or parent-report (CBCL) internalizing or externalizing symptoms. Although in the MLM analysis (Hypothesis 2) there was no *direct* effect of the intervention on depressive symptoms (DIKJ), in the path models the ER strategy cognitive problem-solving (but not self-devaluation or aggressive actions) and the negative parenting subscale of the ESI

revealed significant lagged effects on depressive symptoms (DIKJ). This suggests *indirect* effects of the intervention over time (see estimated path coefficients in Figs. 3 and 4). The intervention had a significant effect on the mediator at T2, and the mediator has a further effect on symptom severity at T3. The intervention also had a significant effect on the mediator at T3, and the mediator has a further effect on symptom severity at T4.

**Cognitive problem-solving** (Fig. 3). Path models indicated that the effect of the intervention on symptoms of depression was mediated by cognitive problem-solving at both T2 and T3. The negative effect of the intervention on cognitive problem-solving at T2 reflects the significant increase in cognitive problem-solving from T1-T2 for the CG but not EG. The positive effect of the intervention on cognitive problem-solving at T3 reflects a greater increase in cognitive problem-solving in the EG (versus CG) from T2 to T3 (not directly tested in Hypothesis 3a). The negative “b” paths imply that increases in cognitive problem-solving were associated with decreases in depressive symptomology.

**Negative parenting style** (Fig. 4**).** Path models indicated that the effect of the intervention on symptoms of depression was also mediated by negative parenting. Although the effect of the intervention on negative parenting at T2 (estimate = -0.09; *p* > .05) and T3 (estimate = -0.12; *p* > .05) individually was not significant, the total increase in negative parenting across both time points was (estimate: − 0.20, *SE* = 0.07, *t* = -2.75, *p* = .006, 95% *CI* [0.345, -0.058]). The negative correlation is consistent with the MLM findings (Hypothesis 3a), suggesting the intervention lead to *reduced* levels of negative parenting. The positive “b” paths indicate that reductions in negative parenting were correlated with reductions in depressive symptoms.

### Aim 4: moderating role of parental depression and stressful life events

#### Stressful life events

Data on the number and impact of stressful life events (CASE; T4) were available for 43 participants. There was no evidence that the *number* or *impact* of negative life events (CASE; T4) moderated the effect of the intervention on children’s depressive symptoms (DIKJ), or self-reported internalizing or externalizing symptoms (YSR) (all *ps* > 0.05). There was some evidence that the effect of the intervention on parent-reported internalizing symptoms (CBCL) at T3 was moderated by the number of negative life events (estimate = 4.12, *SE* = 1.46, *t* = 2.83, *p* = .006, 95% *CI* [1.27, 6.97]). The positive values reflect the fact that EG children who experienced fewer negative life events showed a significant reduction in symptoms (simple slope = -4.878, *SE* = 1.46, *t* = -3.34, *p* = .001, 95% *CI* [-7.86, -1.89]) whereas those with more life events showed no change (*p* > .05). The CG was unaffected by the number of negative life events. There was no evidence that the *impact* of negative life events moderated the effect of the intervention on secondary outcome measures (the models did not converge).

#### Parental depression

Data on the severity of parental depression (BDI-II; T4) were available for 90 parents. There was no evidence that the severity of parental depression or diagnostic status (current versus past depression) at T1 moderated the effect of the intervention on any of the secondary outcome variables (all *ps* > 0.05).

### Aim 5: effects of the intervention on parental depression

Table 4 shows changes in parental depression (BDI-II) from T1-T4 in both groups (EG, CG). MLM with parental depression (BDI-II) as the outcome revealed that both groups show decreased values from T1 to T4 (e = -1.38, *SE* = 0.63, *df* = 72.94, *t* = -2.19, *p* = .031; 95% *CI* [-1.12, -0.87]), but there was no significance difference between EG and CG (e = -0.97, *SE* = 0.87, *df* = 72.43, *t* = -1,11, *p* = .269; 95% *CI* [-1.14, -0.80]). There was no evidence that parental depression changed from T1 to T2 or T3.

**Table 4:** Changes in Parental Depression from T1 to T4 in Both Groups

Experimental Group (*n*, M, SD)				Control group			
T1	T2	T3	T4	T1	T2	T3	T4
*22* 16.64 (9.09)	*22* 12.5 (11.77)	*22* 10.91 (9.22)	*22* 10.05 (9.39)	*18* 21.83 (12.26)	*18* 14.34 (12.11)	*18* 13.89 (11.27)	*18* 14.78 (12.85)

## Discussion

### Summary of findings

The overarching goal of this study was to evaluate the mid-term effects of GuG-Auf, a preventive intervention for children of parents with depression. The German adaptation (“Gug-Auf”) of the Family and Group Cognitive Behavioural (FGCB) [15] intervention has previously shown good acceptability [23] and effectiveness in the short-term [24]. In the current manuscript, statistical analysis of the primary outcome (onset of depression) was not possible due to the small number of cases of depression in both groups. However, we did observe the expected effect of the intervention on the secondary outcome of self-reported internalizing symptoms (a proxy for depression risk) at both T3 and T4. The effect of the intervention on depressive symptoms was mediated by cognitive problem-solving and parenting style. The effects of the intervention on symptoms of psychopathology were not moderated by the severity of parental depression or children’s negative life events.

### Interpretation of findings

Relatively few trials have investigated the extent to which preventive interventions can significantly reduce the *onset of depression* in the offspring of depressed parents. Based on previous studies we had expected roughly 33% of the CG [30] and 10% of the EG [15, 28] to have experienced depression by T4. In fact, just two children across the whole sample had experienced depression by T4, both in the CG. Whilst the number who had been diagnosed with any form of mental disorder by T4 was slightly higher (CG: *n* = 5, 16%; EG: *n* = 2, 6%), it was still too low to allow meaningful statistical comparison between groups. The low overall incidence of mental illness may be partly due to the fact our sample was relatively well-educated and financially-stable, thus having the resources necessary to detect and address early signs of mental illness. However, difficulties coordinating diagnostic interviews with the children 15 months after they had initially been enrolled in the study also contributed to the low numbers.

The fact that we observed an increase in symptom severity over time in the CG reflects the expected risk faced by offspring of depressed parents. This increased risk appears to be “buffered” in the EG, who showed no significant change in internalizing symptoms over time. According to traditional interpretations [55] the size of this effect is “very small”. However, it is worth noting that within the field of youth depression prevention, even the most powerful interventions only reach “small” effects [56]. Furthermore, for interventions such as GuG-Auf, which are ultimately designed to be implemented at a public health level, very small and small effect sizes may translate to large population impact [57]. The robustness of this finding is supported by the fact that the original trial found similar effects of the FGCB intervention [15]. Furthermore, the findings are unlikely to be due to improvements in parents’ own symptoms of depression, since this did not differ between the two groups. Although we would have expected our findings regarding self-reported internalizing symptoms to be mirrored in parents’ reports, this was not the case. This may be due to previously described general discrepancies between parent- and child-reports of internalizing symptoms [43] or specifically as a function of parents’ depression [24]. Of course, it is also possible that children’s self-reports were influenced by a social-desirability effect (knowing what might be expected based on their group allocation). In any case, other studies have found effects to be stronger for children’s self-reports versus parent-reports [18] or clinician reports [30]. The finding that both child- and parent-reports of children’s *externalizing* behaviour did not change in either the EG or CG over time contrasts with previous studies [16, 18] and suggests that the effects of GuG-Auf are specific to internalizing symptoms. This finding is perhaps not surprising given that the intervention does not specifically train children in how to reduce their use of maladaptive strategies such as aggressive behaviour or giving up.

In contrast to the increase in self-reported internalizing symptoms in the CG, we observed a number of positive outcomes for families allocated to the CG. They showed similar improvements to the EG in terms of children’s knowledge of depression and symptoms of depression. Furthermore, the parents in the CG showed similar improvements in their own depressive symptoms to parents in the EG. Given the fact that the sample had a relatively high socioeconomic status and very few children in the CG experienced mental illness across the study period, we believe the improvements in the CG may reflect the resources and psychological resilience and the young age (mean age < 11.59) of our particular sample. It is plausible that once allocated to the CG, these families were motivated to talk about depression within the family or to seek support elsewhere. The discrepancy in findings between changes in internalizing symptoms (YSR) versus depressive symptoms (DIKJ) is hard to interpret. We are somewhat cautious in interpreting the DIKJ values due to relatively poor convergent validity of this measure found in previous studies [24].

A unique aspect of the study design was the inclusion of potentially *mediating* variables. In line with the original trial [28], we found preliminary evidence that the effect of the intervention on depressive symptoms was mediated by cognitive problem-solving and negative parenting, both of which were integral parts of the GuG-Auf intervention. However, our findings warrant some caution in their interpretation. Firstly, cognitive problem-solving and negative parenting mediated the effect of the intervention on depressive symptoms measured using the DIKJ (which has questionable reliability) rather than on internalizing symptoms (which revealed between-group differences in the MLM analyses). Secondly, whilst increased cognitive problem-solving at T3 predicted reduced symptoms of depression at T4, the CG show an initial increase in cognitive problem-solving from T1-T2 which is in the unexpected direction. This may reflect self-initiated attempts to change thought processes as a result of allocation to the CG. The fact that the EG showed no initial change in cognitive problem-solving was unexpected but may indicate that short-term benefits of the intervention are due to non-specific factors (e.g. the group setting). Thirdly, the effects regarding parenting style are restricted to negative (rather than positive) parenting strategies, despite the fact that the intervention involved training positive parenting strategies. Furthermore, although children’s reports of their parents’ parenting arguably carry high ecological validity, we are nevertheless cautious in their interpretation since they may deviate from objective observations or high-frequency measures of self-reported parenting. Although there was evidence that some of the other ER strategies showed between-group differences in their change across time (self-devaluation and aggressive actions), these did not emerge as possible mediators in the path models and were not targeted in the intervention. Other strategies targeted in the intervention (acceptance, distraction, positive thinking, positive activities) showed no change over time. The lack of evidence that attributional style mediated the effect of the intervention on symptoms of psychopathology is perhaps less surprising, given that attributional style was not directly targeted in the intervention. Changes in both groups over time in their knowledge of depression suggest that this is not a unique component of the intervention which contributed to the group differences in internalizing symptoms. Whilst the inclusion of potentially mediating variables was a unique aspect of the study design, we also acknowledge that the sample size of 100 may have been insufficiently powered for mediation analyses. As such, not only may we have failed to detect mediating effects which were present, but the significant effects we did find may be over-estimates of the true effect size. In sum, whilst preliminary evidence suggests the intervention works by modifying cognitions and parenting style, we are cautious in the interpretation of this finding.

We found no evidence that the positive effects of the intervention on internalizing symptoms were *moderated* by parental depression severity, which contrasts with many [12, 17, 30] but not all [18] studies. Since the latter study is the only other to evaluate the FGCB, it is possible that this is evidence of mediated moderation. To our knowledge, this is the first study to investigate the moderating role of negative life events. Unfortunately, we had relatively large amounts of missing data for this variable which limit conclusions. A significant effect was found for the role of the *number* of negative life events on parent-reported internalizing (but not externalizing) symptoms, but since this was not replicated for the *impact* of negative life events on parenting-reported internalizing symptoms, nor for the variables self-reported depressive, internalizing or externalizing symptoms, we refrain from interpreting this finding.

### Strengths and limitations

The main strength of the current study is its clinical and public health relevance: children with a *particularly elevated risk of depression* who received the GuG-Auf intervention showed a significantly *lower risk of internalizing symptoms* in the *mid-term* compared to children who did not receive the intervention. This replication of findings from the original authors of the intervention provides more robust evidence that the intervention is effective at reducing the risk of depression. It is one of few clinical trials to investigate the potential mediators and moderators of preventive interventions for children of depressed parents (see Clinical Implications) and to investigate effects in the mid-term.

A further strength of the study is the use of standardized clinical interviews to assess whether the intervention was effective at preventing the onset of depression. These interviews were also administered to assess the inclusion criteria for the study, meaning that we can be fairly sure that none of the children but all parents in the trial experienced a clinically significant episode of depression and not sub-threshold symptoms or a different disorder altogether. Unfortunately, the number of missing interviews, combined with resilience of the sample, meant that our planned analyses were not possible.

The main limitations of the current study relate to the generalizability of the findings. Firstly, reflecting the enormous strain that many families with a depressed parent experience, there were large amounts of missing data at all four time points. Other trials of preventive interventions for children of parents with depressed have been terminated early, due to the difficulties families with parental mental illness face to commit to even potentially helpful interventions [58]. As we have discussed elsewhere [24] it is unclear whether the observed effects are an over- or under-estimate of the true effect of the intervention. Although we might have expected compliance to be higher in the EG than the CG (who received no intervention), in fact we found the levels of missing data were equal in both groups. Another issue of generalizability is the relatively high socioeconomic status (and lack of ethnic diversity) of the families who participated [24]. Future studies need to do more to address this in terms of recruitment methods (e.g. higher financial rewards for participation) as well as intervention contents (e.g. location at which intervention is delivered).

A second limitation is that despite the intervention targeting communication within families and parent-child relationships, we did not include a measure of family interaction processes. A final limitation is that we did not systematically collect data on what participants in the CG did during the study period. As such it is hard to be sure whether the positive effects seen in the CG reflect a positive effect of the diagnostic sessions at the beginning of the study, or actions families took once allocated to the CG to compensate for not receiving the intervention.

### Clinical implications

As previously mentioned, the positive effects of the intervention at buffering against the increasing risk of depression which children of depressed parents face supports the clinical relevance of the study. GuG-Auf represents an evidence-based intervention which could be integrated into routine healthcare, e.g. in psychiatric clinics for parents suffering from depression. The findings provide a number of insights for the implementation of the intervention at a public health level. Firstly, more development of the intervention may be necessary to ensure it is accessible to a diverse population of families affected by depression. The potentially mediating role of cognitive problem-solving and parenting style suggests that the (cognitive) coping strategies taught to children and the parenting components of the intervention are worthy of retaining in revisions of the intervention. If the intervention is shortened, as suggested by some families who were interviewed [23], contents teaching children about the symptoms and causes of depression might be left out. The findings regarding the lack of moderation by parental depression suggests that the intervention is suitable for delivery in both adult treatment settings as well as non-clinical settings (e.g. parent advice centres).

### Future studies

The difficulty we experienced in recruiting participants for this trial and the large amount of missing data have also been described in other similar trials [58, 59]. This points towards the need for more multi-site trials of preventive interventions. The CHIMPS-Net trial (Clinical Trials Registration DRKS00020380; study protocol in preparation) is an ongoing multi-site trial in Germany designed to evaluate the hurdles to implementing preventive interventions for children of mentally ill parents into routine care. Within this trial the authors are evaluating a modified version of GuG-Auf (“Gug-Auf-Online”), which by shortening the intervention and delivering sessions via video-conferencing aims to reach a more diverse audience [60]. In addition, studies of the cost-effectiveness of the intervention may inform the return of investment by the preventive intervention in the long term.

## Conclusions

This is the first study to evaluate the effects of the FGCB intervention on children’s risk of depression in the mid-term outside of the original research group. We show that the German adaptation GuG-Auf is effective in buffering against the natural increase in self-reported symptoms of internalizing disorders which children of parents with depression show. We found no effects of the intervention on parent-reported internalizing symptoms or externalizing symptoms. Reductions in depressive symptoms are likely to be the result of changes in children’s coping with stress as well as their parents’ parenting. We also observed some improvements in the CG who received no intervention, which may point to the particularly resilient sample in this study. Future endeavours should seek to modify the intervention such that it can be easily accessed on a population level.

## Electronic supplementary material

Below is the link to the electronic supplementary material.

Supplementary Material 1

## Data Availability

The dataset generated and analysed in the current study is available in the Open Science Framework repository, https://osf.io/q7b6r/. The manual is available (in German) from the corresponding author upon reasonable request.

## List of abbreviations

ASF: Attribution Style Questionnaire
BDI: Beck Depression Inventory
CASE: Child and Adolescent Survey of Experiences
CBCL: Child-Behaviour Checklist
CFI: Comparative Fit Index
CG: Control Group
DIKJ: Depression Inventory for Children and Adolescents
DIPS: Diagnostic Interview for Psychiatric Disorders
DSM: Diagnostic and Statistical Manual of Mental Disorders
EG: Experimental Group
ESI: Inventory of Parenting Styles
FEEL-KJ: Emotion Regulation Questionnaire for Children and Adolescents
FGCB: Family Group Cognitive-Behavioural
GuG-Auf: Gesund und glücklich aufwachsen (Growing Up Happy and Healthy)
MLM: Multilevel Modelling
RCT: Randomised Controlled Trial
RMSEA: Root Mean Square of Approximation
SEM: Structural Equation Modelling
YSR: Youth Self-Report

## Footnotes

1 One family was excluded because the parent had clinically-significant values on the SKID-II.

2 We used the identical formulation to the model used here except for the equality constraint that was assumed for the effects of the mediator on the outcome for the T1-T2 and T2-T3 periods (i.e., “b” paths). Information criteria (AIC and BIC) preferred the model with (to that without) this constraint.

3 Some families reported to feel burdened by the numerous questionnaires they were asked to fill in.

4 Between-groups T-tests on data from the oldest child in each family revealed no significant differences at T1 (t1,99 = 0.06; *p* = .415), T2 (t1,99 = 0.17; *p* = .730), T3 (t1,99=-1.95; *p* = .071) or T4 (t1,99 = 0.27; *p* = .786).

5 Because the frequency of psychiatric disorder was so low, we exploratorily investigated whether the frequency of sub-clinical symptoms (mild or elevated) of psychiatric disorder varied between the groups, but again numbers were relatively low (EG = 7, CG = 7). Descriptively we note that anxiety disorders (*n* = 5) were more common than depression (n = 2).

6 There was some evidence that positive thinking increased in the EG from T1 to T3 (estimate = 2.26, *SE* = 0.88, *t* = 2.56, *p* = .012, *d* = 0.38) and remained the same in the CG (estimate = -0.13, *SE* = 0.95, *t* = -0.14, *p* = .891, *d* = − 0.29). However, this difference was not statistically significant (estimate: 2.39, *SE* = 1.30, *t* = 1.84, *p* = .067, *d* = 0.41). There were no significant effects on the other emotion regulation strategies (all *ps* > 0.05).

7 In some models, variances of latent variables did not converge to positive values. This seems to be due to imbalance of the dataset (i.e., missing values) – therefore, we excluded individuals who did not complete the baseline questionnaires; leaving *n* = 87 for these mediation analyses.
