# Supplementary material for: A randomized controlled trial of a preventive intervention for the children of parents with depression: mid-term effects, mediators and moderators
Source: BMC Psychiatry. 2023 Jun 21;23:455. doi: 10.1186/s12888-023-04926-2 (PMC10286417; doi:10.1186/s12888-023-04926-2)
Supplement: Supplementary file 1 — Supplementary Material 1 [file 12888_2023_4926_MOESM1_ESM.docx]

**Supplemental Files**

**Table S1:** *FEEL-KJ emotion regulation strategies and their relation to the GuG-Auf “A-APP” strategies*

|  | **English translations**  **Loechner et al. (2022)** | **Translation authors if different (Cracco et al., 2015)** | **Items used to assess construct** | **GuG-Auf**  **A-APP strategies** |
| --- | --- | --- | --- | --- |
| Adaptive | Problem-solving | Problem-oriented action | -Trying to change what makes me angry*  -Trying myself to make the best of the situation |  |
|  | Distraction |  | -Doing something which I find fun  -Doing something which brings me joy | Positive activities |
|  | Forgetting |  | -Trying to forget what’s making me angry*  -Thinking, that this will pass | Distraction |
|  | Acceptance |  | Making the most of it  Accepting what is making me angry* | Acceptance |
|  | Positive thinking | Humor-enhancement | Thinking about things that make me happy  Remembering nice things | Positive thinking |
|  | Cognitive problem-solving |  | Thinking about how to solve the problem  Thinking about what I can do |  |
|  | Reappraisal | Revaluation | Telling myself it’s not as bad as it seems  Telling myself its nothing important |  |
| Maladaptive | Giving up |  | I don’t want to do anything  I can’t do anything about my anger* anyway |  |
|  | Withdrawal |  | I don’t want to see anyone  Withdrawing myself |  |
|  | Rumination |  | Thinking repeatedly about why I am angry*  Don’t want to let it go from my mind |  |
|  | Self-devaluation |  | Thinking, that it is my problem  Looking for my mistake |  |
|  | Aggressive actions |  | Initiate an argument  Let my feelings out on others |  |

**Table S2:** *Descriptive data on attributional style and knowledge of depression in both groups across all four time points*

|  | Experimental Group  *n*, M, (SD) | | | | | | | | | Control Group  *n*, M, (SD) | | | | | | | |
| --- | --- | --- | --- | --- | --- | --- | --- | --- | --- | --- | --- | --- | --- | --- | --- | --- | --- |
|  | T1 | |  | T2 |  | T3 |  | T4 |  | T1 |  | T2 |  | T3 |  | T4 |  |
| Positive attributional style (ASF) |  |  | |  |  |  |  |  |  |  |  |  |  |  |  |  |  |
| Internal | *56* | 22.32  (3.44) | | *43* | 22.33  (3.25) | *40* | 22.15  (3.4) | *40* | 22.5  (2.98) | *48* | 23.13  (3.59) | *35* | 23.43  (3.07) | *39* | 22.74  (3.6) | *38* | 23.63  (2.53) |
| Stability | *56* | 24.54  (4.33) | | *43* | 25.72  (4.08) | *40* | 25.53  (4.31) | *40* | 24.93  (4.07) | *48* | 23.9  (5.09) | *35* | 25.06  (4.21) | *39* | 24.31  (5.33) | *38* | 23.18  (5.19) |
| Globality | *56* | 21.34  (4.41) | | *43* | 22.67  (4.08) | *40* | 22.65  (4.17) | *40* | 21.78  (4.87) | *48* | 20.88  (5.46) | *35* | 22.83  (4.38) | *39* | 21.13  (4.66) | *38* | 21.32  (5.04) |
| Negative attributional style (ASF) |  |  | |  |  |  |  |  |  |  |  |  |  |  |  |  |  |
| Internal | *56* | 20.41  (3.27) | | *43* | 20.91  (3.23) | *40* | 20.73  (2.82) | *40* | 21.23  (3.44) | *48* | 21.42  (4.54) | *35* | 22.54  (2.98) | *39* | 21.03  (2.84) | *38* | 21.37  (3.09) |
| Stability | *56* | 22.80  (4.47) | | *43* | 23.16  (4.66) | *40* | 23.65  (5.09) | *40* | 22.68  (4.34) | *48* | 21.23  (5.82) | *35* | 23.31  (5.83) | *39* | 22.05  (6.03) | *38* | 20.87  (5.57) |
| Globality | *56* | 18.84  (5.21) | | *43* | 20.44  (4.69) | *40* | 21  (4.97) | *40* | 19.95  (5.49) | *48* | 18.27  (5.29) | *35* | 20.14  (5.33) | *39* | 19.44  (5.58) | *38* | 19.37  (5.45) |
| Knowledge of Depression | *41* | 33.85 (4.54) | | *34* | 35.94 (2.90) | *30* | 36.33 (6.42) | *24* | 35.45 (3.29) | *34* | 32.64 (3.44) | *27* | 34.55 (3.90) | *29* | 34.83 (3.01) | *26* | 35.11 (3.19) |
|  |  |  | |  |  |  |  |  |  |  |  |  |  |  |  |  |  |

**Table S3**. Medians, IQRs, and Skewness of Depression Measures.

|  |  | Control |  |  | Experimental | |  |
| --- | --- | --- | --- | --- | --- | --- | --- |
|  |  | Median | IQR | skew | Median | IQR | skew |
| Depressive symptoms (DIKJ) | T1 | 6 | 8 | 0.6 | 7 | 5.25 | 1.5 |
|  | T2 | 5 | 7 | 0.9 | 7 | 7.5 | 2.0 |
|  | T3 | 5 | 4 | 1.4 | 5 | 7.5 | 2.0 |
|  | T4 | 3 | 5 | 1.8 | 5 | 7 | 1.1 |
| Parent-report internalizing (CBCL) | T1 | 6 | 8 | 1.0 | 6 | 10 | 0.7 |
|  | T2 | 4 | 9 | 1.5 | 4 | 6 | 2.0 |
|  | T3 | 5 | 7.25 | 0.8 | 3 | 6 | 1.5 |
|  | T4 | 6 | 7 | 1.5 | 3 | 5.5 | 1.6 |
| Parent-report externalising (CBCL) | T1 | 4 | 4.5 | 3.1 | 5 | 8 | 1.8 |
|  | T2 | 2 | 5 | 1.6 | 5 | 5.5 | 2.1 |
|  | T3 | 2.5 | 4 | 1.1 | 4 | 7 | 1.5 |
|  | T4 | 2 | 6 | 0.4 | 3 | 6.5 | 1.5 |
| Self-report internalizing (YSR) | T1 | 5.5 | 7.5 | 0.9 | 7 | 9.75 | 1.3 |
|  | T2 | 6 | 10.5 | 0.8 | 5.5 | 6 | 2.6 |
|  | T3 | 8 | 10 | 1.4 | 6 | 4.25 | 1.9 |
|  | T4 | 6.5 | 8.5 | 2.0 | 4 | 6 | 2.4 |
| Self-report externalising (YSR) | T1 | 7 | 8 | 1.2 | 8 | 7.5 | 0.6 |
|  | T2 | 8 | 8.5 | 0.6 | 7 | 7.75 | 1.5 |
|  | T3 | 7 | 9 | 0.9 | 6.5 | 6.25 | 1.0 |
|  | T4 | 7 | 10 | 0.5 | 6.5 | 6.5 | 1.1 |

Note. Skewness < -2 or > 2 may indicate non-normality

**Table S4**: Frequency of missing values between groups across time

|  |  | BDI-II  parents | DIKJ | CBCL | YSR | FEEL-KJ | ASF | Knowledge of Depression | ESI |
| --- | --- | --- | --- | --- | --- | --- | --- | --- | --- |
| T1 | EG | 2 | 11 | 8 | 9 | 6 | 17 | 9 | 14 |
|  | CG | 8 | 17 | 16 | 17 | 13 | 21 | 16 | 21 |
| T2 | EG | 18 | 24 | 20 | 16 | 18 | 28 | 16 | 28 |
|  | CG | 24 | 29 | 24 | 24 | 24 | 29 | 23 | 29 |
| T3 | EG | 19 | 18 | 18 | 19 | 18 | 19 | 20 | 18 |
|  | CG | 26 | 28 | 23 | 23 | 20 | 20 | 21 | 23 |
| T4 | EG | 41 | 24 | 24 | 24 | 24 | 24 | 26 | 24 |
|  | CG | 37 | 26 | 24 | 25 | 23 | 23 | 24 | 23 |

*Note.* BDI-II = Beck’s Depression Inventory; DIKJ = Depressions-Inventar für Kinder und Jugendliche; CBCL = Child Behaviour Checklist; YSR = Youth Self-Report; FEEL-KJ = Fragebogen zur Erhebung der Emotionsregulation bei Kindern und Jugendlichen; ASF = Attributionsstil-Fragebogen für Kinder und Jugendliche; ESI = Erziehungsstil-Inventar.

**Figure S1:** *Changes in Cognitive Problem-Solving in both Groups over Time*

**Figure S2:** *Changes in Self-Devaluation in both Groups over Time*

**Figure S3:** *Changes in Aggressive Actions in both Groups over Time*
